# Traditional Chinese Medicine for HIV-Associated Acute Herpes Zoster: A Systematic Review and Meta-Analysis of Randomized Trials

**DOI:** 10.1155/2022/8674648

**Published:** 2022-02-18

**Authors:** Yue Jiang, Ruo-Xiang Zheng, Ze-Yu Yu, Xiao-Wen Zhang, Jing Li, Hui-Di Lan, Shu-Yu Qiao, Mei Han, Hui-Juan Cao, Nicola Robinson, Jian-Ping Liu

**Affiliations:** ^1^Centre for Evidence-Based Chinese Medicine, Beijing University of Chinese Medicine, No. 11 Bei San Huan Dong Lu, Chaoyang District, Beijing 100029, China; ^2^Beijing Key Laboratory of the Innovative Development of Functional Staple and the Nutritional Intervention for Chronic Disease, China National Research Institute of Food & Fermentation Industries Co, Ltd, Beijing 100015, China; ^3^School of Traditional Medicine, Guangxi University of Chinese Medicine, No. 13 Wu He Da Dao, Nanning, Guangxi 530000, China; ^4^Institute of Health and Social Care, London South Bank University, 103 Borough Road, London, UK

## Abstract

**Background:**

Herpes zoster (HZ) is a common infection in individuals with acquired immunodeficiency syndrome (AIDS) patients. Traditional Chinese medicine (TCM) has been used widely in clinical practice for HZ, which remains not supportive of evidence. This review aimed to evaluate the effectiveness and safety of TCM in treating HIV-associated HZ.

**Methods:**

Nine electronic databases were searched for randomized controlled trials (RCTs) testing TCM in treating HIV-associated HZ. Data were extracted on citations, interventions, and outcomes, by two authors independently. For the quality evaluation, Cochrane risk-of-bias tool 2.0 was used. Meta-analyses were performed by Revman5.3 software. Effect estimation presented as risk ratio (RR) for dichotomous data and mean difference (MD) for continuous data with their 95% confidence interval (CI).

**Results:**

Twelve RCTs (*n* = 644) were included; the majority of them had a high or unclear risk of bias. Meta-analysis showed that pain intensity (VAS 0–5) in the Chinese herbal medicine (CHM) group was lower than it in the drugs group (MD = −0.87, 95% CI [−1.69, −0.04], two trials, *n* = 93). Duration of herpes-related pain (days) of patients in the combination group was shorter than those in the drugs group (MD = −9.19, 95% CI [−16.73, −1.65], *n* = 144). The incidence of postherpetic neuralgia (PHN) in the combination group was lower than in the drugs group (RR = 0.49, 95% CI [0.25, 0.99], *n* = 202). As for cure rate (complete absence of pain and herpes), two trials showed that CHM was better than drugs (RR = 1.58, 95% CI [1.13, 2.22], *n* = 93), five trials showed combination treatment was better than drugs (RR = 1.40, 95% CI [1.08, 1.82], *n* = 224). The cure rate in the acupuncture group was more than that in the drugs group (RR = 1.99, 95% CI [1.18, 3.36], *n* = 120). Four trials reported adverse effects and found no serious adverse events occurred.

**Conclusion:**

CHM and acupuncture demonstrate more benefits than drugs in pain relief, cure rate improvement, and incidence reduction of PHN. However, given the data limitation and TCM therapies' diversity, the conclusions need to be verified in future trials.

## 1. Background

Acquired immunodeficiency syndrome (AIDS) is an autoimmune disease caused by the human immunodeficiency virus (HIV). HIV destroys the human immune system, attacks helper T cells, infects B cells, and significantly decreases peripheral blood lymphocytes. Highly active antiretroviral therapy (HAART) is widely used for HIV infection. The treatment can effectively inhibit the replication of HIV transcription and reduce AIDS mortality; however, it can also lead to decreasing of CD4 + T cells and damage of the kidney and liver [1–3].

Herpes zoster, an acute infectious skin disease caused by the varicella-zoster virus (VZV), usually occurs between the earlier HIV infection stage and the period of AIDS [4]. The virus invades and is latent along the sensory nerve. When the body's immune function is weak, the latent virus replicates and spreads, resulting in HZ [5, 6]. For patients with AIDS, HZ is more likely to occur and bring great pain due to its positron neuralgia, which greatly affects the quality of life [7, 8]. The main drugs recommended in the China Guideline on the Diagnosis and Treatment of AIDS for HIV complicated with HZ include acyclovir, famciclovir, and valaciclovir [9].

Nevertheless, a series of reports remind us that acyclovir may lead to renal damage [10], and the use of ganciclovir in treating HZ may be controversial [11]. Due to intolerant pain, many patients seek traditional Chinese medicine (TCM) for relieving symptoms. TCM therapies often include herbal medicine (topical use and oral taking), acupuncture, and cupping. Since there have been a substantial number of clinical studies published, we, therefore, evaluated current clinical evidence from randomized trials on the effectiveness and safety of TCM in the treatment of HIV-associated herpes zoster.

## 2. Methods

This meta-analysis was performed following the PRISMA 2020 guidelines for systematic reviews and meta-analyses [12].

### 2.1. Inclusion/Exclusion Criteria

#### 2.1.1. Study Type

Randomized controlled trials (RCTs) were included without restriction on language, publication type, or blinding.

#### 2.1.2. Participants

Patients infected with HIV or diagnosed as AIDS and complicated with acute HZ at the same time will meet the main inclusion criterion, without the restriction on age, race, and nationality. But the duration of HZ should be less than three weeks [13].

#### 2.1.3. Interventions

Trials testing any TCM therapies such as Chinese herbal medicine (CHM) or acupuncture, moxibustion, and cupping with or without drugs (e.g., antiviral drugs, neurotrophic drugs, painkillers, and other common symptomatic treatments) were included. A combination of different TCM therapies as experimental treatment was also included.

#### 2.1.4. Controls

The controls included no treatment, placebo, or drugs therapy. If there were any drug therapy in the control group, it must stay the same as the intervention group.

#### 2.1.5. Cointerventions

If there were any cointerventions for HIV/AIDS, such as HAART or active antiretroviral therapy (ART), it should be the same between the control and intervention groups.

#### 2.1.6. Outcomes

1. Primary outcomes: indicators related to pain including pain intensity (VAS score and McGill score) and duration of pain, incidence of postherpetic neuralgia (PHN), cure rate by the end of treatment, cure defined as a complete absence of pain and herpes, and cure rate defined as the number of cured/total number*∗*100% [14]
2. Secondary outcomes: quality of life (World Health Organization Quality of Life – WHOQOL and Karnofsky Performance Status – KPS) and adverse events

### 2.2. Searching Strategies

Up to 23 May 2021, we systematically searched the following electronic bibliographic databases: China National Knowledge Infrastructure (CNKI), Wanfang Database, Chong Qing VIP, SinoMed, PubMed, Embase, the Cochrane Library, WHO International Clinical Trial Registration Platform (WHO ICTRP), and https://ClinicalTrials.gov. The details of searching strategies are shown in Additional file 1.

### 2.3. Screening

All retrieved trials were managed using NoteExpress software. Initial screening was carried out based on inclusion/exclusion criteria after reading article titles and abstracts. The full-text screening was acquired and checked for eligibility before the final analysis. Two authors carried out back-to-back literature screening independently. Any disagreement was solved through discussions and consultation with a third author.

### 2.4. Data Extraction

The extracted data were: (1) basic characteristics of included trials (first author, research topic, year of publication, etc.); (2) diagnosis information; (3) inclusion and exclusion criteria; (4) baseline characteristics; (5) control and intervention details; (6) primary outcome and their definitions, measuring time points, and follow-up; and (7) risk of bias components.

### 2.5. Quality Evaluation

The methodological quality of included trials was evaluated by the risk of bias assessment tool 2.0 [15] developed by Cochrane Collaboration. This tool assesses the risk of bias from five domains: randomization process, intended interventions, missing data, outcome measurement, and selective reporting (valid/invalid). Only if there are five domains assessed as low, the overall risk is low; otherwise, even if one domain considered as some concern/high, the overall risk will turn in some concern/high. And if there were four or more domains assessed as some concerns, the overall risk will turn high.

Two authors (Y Jiang and RX Zheng) independently extracted data and assessed and verified the risk of bias. The results were cross-referenced, and any differences were resolved through discussion with the third author (J Li or JP Liu).

### 2.6. Data Analysis

Review Manager 5.3 software [16] was used for data pooling and statistical analysis. According to the inclusion criteria of intervention and control, trials were divided into four comparisons. Meta-analysis was conducted for each comparison according to different outcomes. Mean difference (MD) and 95% confidence interval (CI) were used for continuous outcomes, and relative risk (RR) was calculated with 95% CI for dichotomous outcomes.

The Cochran Q test and the *I*^2^ statistics were used to examine the heterogeneity across the trials. The random-effects model was used to estimate the size of the combined effects in the meta-analyses.

If *P* < 0.01 and the source of heterogeneity was unknown, descriptive analysis was used instead of meta-analysis. Subgroup analyses were conducted by different comparisons, such as CHM versus drugs, CHM + drugs versus drugs, acupuncture and moxibustion versus drugs, and so on, if the number of included studies is sufficient.

Due to the large variability of TCM and the different combination of TCM and drugs, ineradicable clinical heterogeneity of the treatment measures in the included studies, the random-effects model was used to combine the data. The related charts for meta-analysis should be carried out, such as forest and funnel plots.

## 3. Results

### 3.1. Study Search

**Figure 1:**
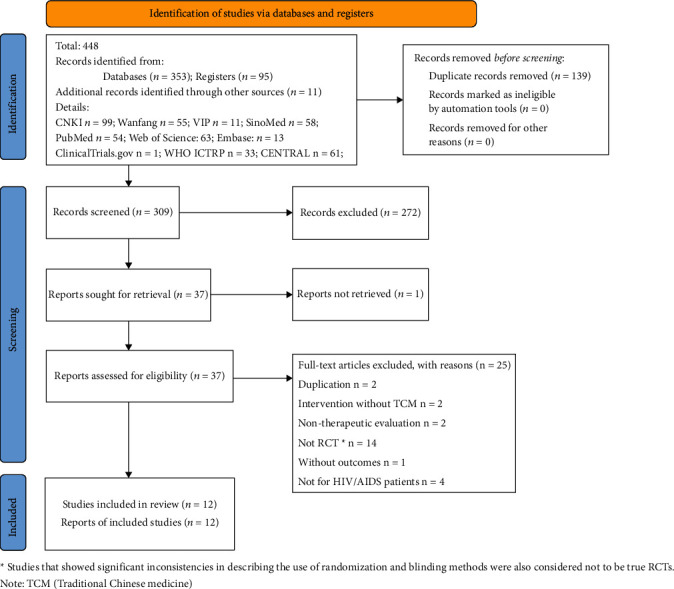
Flowchart of study searches and screening. Note: TCM – traditional Chinese medicine.

In total, 448 trials (223 in Chinese and 225 in English) were searched; 139 were excluded before screening due to duplication; 272 were excluded at titles-abstracts screening; and 25 were excluded at full-text screening. Eventually, 12 trials were included in our review. The inclusion process and exclusion reasons for screening are shown in Figure 1.

### 3.2. Characteristics of the Included Trials

The general information of included trial is presented in Table 1. The trials were published between 2000 and 2014 and were conducted in China. The participants in the trials were mainly male, while only three trials were female-dominated [18, 21, 28]. There were 644 participants: 348 in the control group and 196 in the intervention group. Only one trial [26] reported cointervention as HAART but no further details.

According to the intervention and controls, trials were divided into three comparisons: (1) CHM versus drugs (two trials), (2) CHM + drugs versus drugs (eight trials) [20–27], and (3) acupuncture versus drugs (two trials) [28, 29].

The baseline information of patients in included trials is shown in Table 1.

TCM therapies contain oral taken herbal granules, herbal medicine for external use, Chinese herbal injection and acupuncture, or moxibustion. Drugs included antiviral agents, nonsteroidal antipyretic and painkillers, neurotrophic agents, and painkillers with the administration as injection, oral use, and external use (Table 2).

### 3.3. Methodological Quality

**Figure 2:**
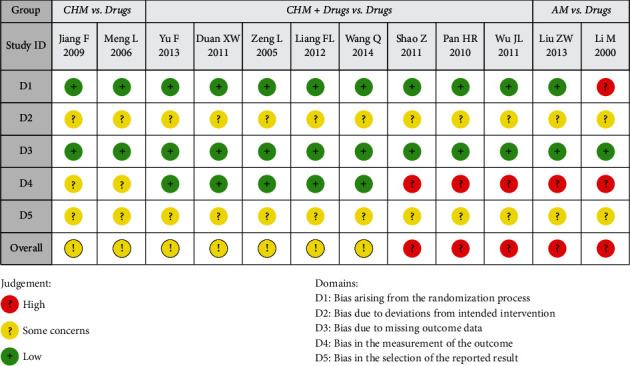
Risk of bias assessment of including trials using Rob2 tool. Domains: D1: bias arising from the randomization process, D2: bias due to deviations from intended intervention, D3: bias due to missing outcome data, D4: bias in the measurement of the outcome, and D5: bias in the selection of the reported result.

Among 12 included trials, none had a low risk of bias. Seven trials had some concern of risk of bias, and the other five trials had a high risk of bias. Details regarding downgrading are provided in Figure 2. Although four studies [19, 21–23, 25, 27] did not specify randomization, from the same group with the studies that did specify randomization [17, 18, 20, 24, 26]. Thus, researchers identified these ten trials with a low risk of bias in domain 1 (Additional file 4-1). We could not get access to the trial protocols, and no trial indicated registration information. Therefore, we evaluated deviations from the intended interventions (domain 2, Additional file 4-2) with some concern. No trials reported missing data, and the number of randomized participants was consistent with that in statistical analyses. Thus, all trials were identified with low in domain 3 (Additional file 4-3). No objective outcomes were used in seven trials [17, 18, 24–28], and the outcome measurement is likely to be influenced by lack of blinding. Domain 4 in seven trials [17, 18, 24–28] were identified with high (Additional file 4-4). No registered protocol of the included trials was mentioned, so the authors determined to evaluate the selective bias of other trials with some concern (domain 5, Additional file 4-5; Figure 2). And the percentage figure for risk of bias is presented in Additional file 3.

### 3.4. Analysis of Overall Effects

Data from ten trials were included in meta-analyses, focusing on five classes of outcomes, and the results are shown as follows.

#### 3.4.1. Pain

Four trials from three comparisons reported pain scores (VAS, scale of 1 to 5). One trial [27] showed pain in acupuncture, and moxibustion combined with CHM (external use) was lower than that in drugs (MD −1.10, 95% CI [−1.70, −0.50]). Two trials [17, 18] showed more significant pain relief in the CHM group than in the drugs group (MD −0.87, 95% CI [−1.69, −0.04]). One trial [25] showed no difference in pain relief between CHM wash lotion + drugs and drugs (MD = −0.47, 95% CI [−1.38, 0.44]; Table 3).

Duration of pain: Five trials from two comparisons reported time of pain; it showed a significant reduction in CHM + drugs group in the duration of pain (MD −9.19 days, 95% CI [−16.73, −1.65], four trials), then in drugs group [20, 23–25]. And in the acupuncture and moxibustion + drugs group, the reduction in the duration of pain was also more than in the drugs group (MD −5.87 days, 95% CI [−7.85, −3.89], one trial) [27] (Table 3).

#### 3.4.2. Incidence of Postherpetic Neuralgina

Three trials [19, 22, 23] reported the incidence of PHN and showed a benefit of CHM + drugs in reducing the incidence of PHN (RR 0.49, 95% CI [0.25, 0.99]), compared with drugs (Table 3).

#### 3.4.3. The Cure Rate of Herpes Zoster

**Figure 3:**
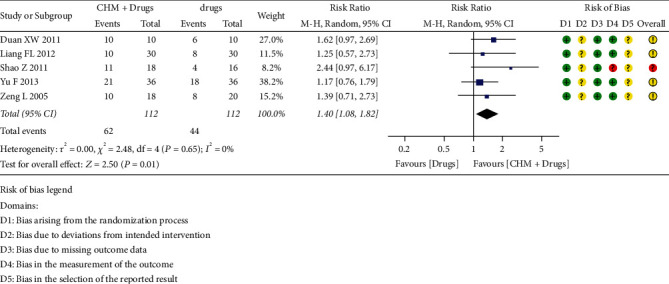
Cure rate of HZ for CHM + drugs versus drugs.

Nine trials exhibited significantly better cure rate in CHM, compared with drugs (RR 1.58, 95% CI [1.13, 2.22], two trials) [17, 18]; better cure rate in CHM + drugs, compared with drugs (RR 1.40, 95% CI [1.08, 1.82], five trials) [19–22, 24]; and better cure rate in acupuncture and moxibustion, compared with drugs (RR 1.99, 95% CI [1.18, 3.36], two trials) [27, 28] (Figure 3).

#### 3.4.4. Quality of Life

Two trials [24, 26] reported the quality of life, measured by the WHOQOL-100, from 1 to 100, and it showed that CHM + drugs improved quality of life than drugs (MD = 4.72 scores, 95% CI [0.45, 8.98]; Table 3).

### 3.5. Adverse Effects

#### 3.5.1. Four Trials Reported Adverse Effects [17, 18, 23, 24]

Two trials compared CHM with drugs, and both treated intervention group with Long Dan Xie Gan granules + Ruyi Jinhuang paste and control group with acyclovir. One of them [17] reported no drug-related adverse reaction. The other one [18] said a few cases underwent abnormal liver function, but not an exact number, with no abnormal index in routine blood, urine, and kidney function test.

Two trials compared CHM + drugs with drugs. One trial [24] reported no drug-related adverse reaction. Intervention treatment of this trial was Long Dan Xie Gan granules or Ba Zhen formula + ganciclovir + unknown AA for external use + compound glycyrrhizin injection + BCG-PSN + vitamin B12 + calamine. At the same time, the control group was treated with all the same as the intervention group except CHM. One trial [23] reported three cases of slightly dizzy (intervention group: two cases and control group: one case) after the first dose of intravenous antiviral drugs but all of the cases with no abnormal index in routine blood, urine, liver function, and kidney function test. The intervention of this trial is with Long Dan Xie Gan formula or Chu Shi Wei Ling formula + Sanhuang wash lotion + valacyclovir + acyclovir, and the control is with valacyclovir + acyclovir.

## 4. Discussion

### 4.1. Main Finding

Sixteen trials of HIV-associated herpes zoster were found, but 4 trials focused on postherpetic neuralgia. Only 12 RCTs (*n* = 644) focused on acute herpes zoster were included in this review. Those trials represent both genders and cover all ages. All trials were published between 2000 and 2014 and were conducted in China. CM treatment includes oral taken granules of CHM, external use CHM, injection of CHM and acupuncture, and moxibustion. Due to the limitation and the small sample size of each included trial, we cannot draw firm conclusions relating TCM therapy in treating patients with HIV-associated acute herpes zoster.

Compared with drugs, CHM + external use demonstrated positive effects in improving cure rate and alleviating pain (VAS). CHM + drugs are much better than drugs on improving cure rate, shortening the duration of pain, reducing the incidence of PHN, and improving cure rate and quality of life (WHOQOL-100) at the end of treatment. Acupuncture and moxibustion demonstrated positive effects in alleviating pain (VAS), shortening the duration of pain, and improving the cure rate at the end of treatment. Four included trials reported the outcome of adverse events; two reported nonserious adverse events.

### 4.2. Comparison with Other Studies

The correlative factor for HIV-associated HZ is usually around hypoimmunity and immunosuppression, which are hard to fix. Compared with drugs, four systematic reviews suggested positive effects of TCM therapy. Wet cupping [29], Long Dan Cao (*Gentiana scabra* Bunge.) single herb formula [30], and acupuncture [31] may decrease the rate of PHN. Acupuncture using independently [31] and acupuncture plus moxibustion [32] may reduce the VAS score of pain. *Gentiana scabra* Bunge. formula (Long Dan Cao single herb formula) could also shorten the pain duration [30].

The clinical significance of those study results is yet to be established, and high-quality evidence with rigorous research methods is still insufficient.

### 4.3. Limitations

The measures of the control group of integrative medicine were inconsistent, which might be because of the unregulated drug use. And the number of included trials and the number of sample sizes were small; the trial scale was limited; and the general quality was low. Most of the included trials did not describe the specific randomization method, did not use the blind method, and evaluated outcome indicators mainly consisted of doctor evaluation or patient self-rating scale, which was highly subjective. Therefore, the meta-analysis results can only indicate the partial effect of CHM and AM on HIV-associated HZ, and the reliability of the conclusions obtained is limited.

### 4.4. Implications for Clinical Practice and Further Research

Our review suggests that TCM may be a potential alternative therapy for the treatment of HIV-associated HZ. Still, most of the included trials have modified the original formula regimen of CHM. Therefore, clinicians should add and subtract some herbs according to physical signs in clinical practice to improve clinical symptoms.

The included trials were limited by the small sample size and relatively low methodological quality. We hope that in the future, with the support of a more rigorous methodology, more multicenter clinical trials of CHM treatment of HIV/AIDS-associated HZ will be carried out with large sample size, especially RCT trials. More high-quality evidence will help verify the effectiveness of CM and integrative medicine treatment and provide more reliable support for this clinical problem.

In terms of basic research, the research on the components of CHM, the effective mechanism of acupuncture and moxibustion therapy, and the interaction between Chinese and drugs still need to be further explored to provide an experimental basis for clinical research on this topic.

## 5. Conclusion

Low certainty of evidence showed that Chinese herbal remedies and acupuncture might be beneficial in relieving pain for HIV-associated HZ and appears to be safe. Further research with a higher quality of evidence is still needed before the clinical recommendation of TCM therapies.

## Figures and Tables

**Table 1:** Characteristics of included trials on traditional Chinese medicine for herpes zoster.

Trial ID	Sample I/C	Gender M/F	State of HIV	Age (years)	Treatment regimen	Duration	Outcomes
CHM vs. drugs							
Jiang F, 2009 [17]	30/30	I: 19/11	HIV/AIDS	I: 41.3 ± 7.1	I: Long Dan Xie Gan granules (6 g/po./tid.) + Ruyi Jinhuang paste (external use, q.s./p.a.a./bid. or tid.)	14 d	Pain (VAS score); cure rate; adverse effects
		C: 17/13		C: 45.3 ± 8.7	C: acyclovir (0.2 g/po./5 times per day) + acyclovir ointment external (q.s./p.a.a./bid. or tid.)		
Meng L, 2006 [18]	15/18	I: 10/16	HIV/AIDS	I: 42.5 ± 7.5	I: Long Dan Xie Gan granules (6 g/po./tid.) + Ruyi Jinhuang paste (external use, q.s./p.a.a./bid. or tid.)	14 d	Pain (VAS score); cure rate; adverse effects
		C: 11/18		C: 47.7 ± 9.2	C: acyclovir (0.2 g/po./5 times per day) + acyclovir ointment external (q.s./p.a.a./bid. or tid.)		
CHM + drugs vs. drugs							
Yu F, 2013 [19]	36/36	55/17	HIV/AIDS	I: 60.2 (50∼81)	I: Tanreqing injection (20 ml/5% GS 250 ml ivgtt./qd.) + CHM wash lotion (1,000∼2,000 mL/p.a.a. for 20∼25 min/bid.) + (acyclovir + acyclovir ointment external + diclofenac sodium and carbamazepine) (same as control)	21 d	Incidence of PHN; cure rate
				C:/	C: acyclovir (5∼10 (mg/kg)/5% GS 250 ml vgtt./q8h.) + acyclovir ointment external (q.s./p.a.a./unknown) + diclofenac sodium and carbamazepine (discretion/unknown/unknown)		
Duan XW, 2011 [20]	10/10	14/6	AIDS	37.34 ± 12.9	I: Long Dan Xie Gan granules (unknown/po./bid.) + valacyclovir (200 mg/po./bid.)	45 d	Pain time (days); cure rate
					C: valacyclovir (200 mg/po./bid.)		
Zeng L, 2005 [21]	18/20	I: 6/12	AIDS	I: 44.6 (36∼52)	I: Long Dan Xie Gan formula (elixation/po./bid.) + jidesheng sheyao tablets (external; grind into powder and mix with water to make a paste, q.s./p.a.a./q3h) + (acyclovir + vitamin B1 and vitamin B12 + somedon) (same as control)	7 d	Cure rate
		C: 9/11		C: 42.6 (28∼50)	C: acyclovir (10 mg/(kg)/ivgtt./q8h) + vitamin B1 and vitamin B12 (unknown/po./unknown) + somedon (p.r.n./po./unknown)		
Liang FL, 2012 [22]	30/30	41/19	HIV/AIDS	67.2 (60∼81)	I: herbal gargle (add 500 mL water boil to 250 mL elixation/p.a.a. 20∼25 min/bid.; Patients with oral herpes: 30–50 ml/Garg.3∼5 min/bid.) + (acyclovir + ibuprofen or tramadol) (same as control)	10–30 d	Incidence of PHN; cure rate
					C: acyclovir (5∼10 mg/kg/ivgtt./q8h.) + ibuprofen or tramadol (p.r.n./po./unknown)		

Wang Q, 2014 [23]	35/35	I: 18/17	AIDS	45 ± 2	I: Long Dan Xie Gan formula or Ba Zhen formula (elixation/po./bid.) + (ganciclovir + unknown AA for external use + compound glycyrrhizin injection + BCG-PSN + vitamin B12 + calamine) (same as control)	14–21 d	Pain time (days); incidence of PHN; adverse effects
		C: 18/17			C: ganciclovir (0.5 g/ivgtt./q8h.) + unknown antiviral agent for external use (q.s./p.a.a./unknown) + compound glycyrrhizin injection (40 mL/5% GS. Unknowm/Qd. 7 times) + BCG-PSN (1 mg/im./qod. for 4 times) + vitamin B12 (q.s./po./unknown) + calamine (p.r.n./p.a.a./unknown)		
Shao Z, 2011 [24]	18/16	I: 17/1	HIV/AIDS	I: 35.78 ± 7.605	I: Long Dan Xie Gan formula or Chu Shi Wei Ling formula (elixation half dose/po./bid.) + Sanhuang wash lotion (q.s./po./unknown) + (valacyclovir + acyclovir) (same as control)	28 d	Pain (VAS score); cure rate; quality of life (WHOQOL); adverse effects
		C: 14/2		C: 35.88 ± 7.940	C: valacyclovir (300 mg/po./bid) + acyclovir (5–10 (mg/kg)/unknown/q8h.)		
Pan HR, 2010 [25]	10/10	I: 9/1	HIV/AIDS	I: 20∼35: *n* = 5, 36∼50: *n* = 3, ≥51: *n* = 2	I: Long Dan Xie Gan formula or Chu Shi Wei Ling formula (elixation half dose/po./bid.) + Sanhuang wash lotion (q.s./po./unknown) + (valacyclovir + acyclovir) (same as control)	28 d	Pain time (days)
		C: 6/4		C: 20∼35: *n* = 3, 36∼50: *n* = 4, ≥51: *n* = 3	C: valacyclovir + acyclovir (300 mg/po./bid) + acyclovir (5–10 (mg/kg)/unknown/q8h.)		
Wu JL, 2011^*∗*^ [26]	76/41	I: 57/17	AIDS	I: 40.12 (26∼68)	I: Long Dan Xie Gan formula or Chu Shi Wei Ling formula (elixation/unknown/unknown) + (valacyclovir + acyclovir) (same as control)	24 d	Quality of life (WHOQOL)
		C: 29/12		C: 38.98 (22∼66)	C: valacyclovir + acyclovir (300 mg/po./bid) + acyclovir (5–10 (mg/kg)/unknown/q8h.)		
Acupuncture vs. drugs							
Liu ZW, 2013 [27]	30/30	I: 17/13	HIV/AIDS	I: 44 ± 21	I: acupuncture (encircling needling/0.38 mm*∗*25 mm, 15°/20 min) + thread-moxa in Zhuang folk medicine (thread-moxa/0.7 mm*∗*300 mm/sparkle press acupoint for 2∼3 times) + jingwanhong burn ointment (external, q.s./po./after thread-Moxa) C: nimesulide (0.1 g/p.o./bid.) + valacyclovir (250 mg/p.o/tid.) + vitamin B1 (20 mg/po./tid.) + ribavirin ointment external (q.s./p.a.a./qid.)	14 d	Pain (VAS score); cure rate
		C: 16/14		C: 46 ± 18			
Li M, 2000 [28]	20/40	50/60	HIV/AIDS	(20∼50)	I: moxibustion (regular moxibustion: unknown/31 min) + acupuncture (blood-letting puncture: three-edged needle/several drop of blood reducing acupuncture method: 0.38 mm*∗*25 mm, 15°/30 min. Encircling needling + reducing acupuncture method: 0.38 mm*∗*25 mm, 15°/30 min; transverse needling: 0.38 mm*∗*25 mm, 15°/30 min) C: acyclovir (200 mg/p.o/tid.) + acyclovir external (q.s./p.a.a./tid.)	27 d	Cure rate

Note: I: intervention group, C: control group, M: male, F: female, CHM: Chinese herbal medicine; and the cure rate of HZ (cure: the complete absence of pain and herpes; cure rate: number of cured/total number × 100%). ^*∗*^Wu JL, 2011, is the only trial reported cointervention: HAART, with no further details. C:/ means that the age data of the control group in Yu F, 2013 were unreported.

**Table 2:** Details of the TCM therapies of included trials.

Study ID	Name of TCM therapy and delivery (dosage for 1 use/usage/frequency)	Composition of CHM
Jiang F, 2009 [18]	Long Dan Xie Gan granules: 6 g/po./tid	Long Dan Xie Gan granules: *Gentianae Radix et Rhizoma* (longdan), *Gardeniae Fructus* (zhizi), *Scutellariae Radix* (huangqin), *Bupleuri Radix* (chaihu), *Rehmanniae Radix* (shengdi), *Plant Aginis Semen* (cheqianzi-with hot salt frying), *Alismatis Rhizoma* (zexie), *Aristolochia Manshuriensis* (guanmutong), *Angelicae Sinensis Radix* (danggui-with hot alcohol frying), and *Glycyrrhizae Radix et Rhizoma Praeparata Cum Melle* (zhigancao)
	Ruyi Jinhuang paste (external): q.s./p.a.a./bid. or tid	Ruyi Jinhuang paste (external): *Trichosanthis Radix* (tianhuafen), *Curcumae Longae Rhizoma* (jianghuang), *Paeoniae Radix Alba* (baizhi), *Atractylodis Rhizoma* (cangzhu), *Arisaemants Rhizoma* (tiannanxing), *Glycyrrhizae Radix et Rhizoma* (gancao), and *Rhei Radix et Rhizoma*

Meng L, 2006 [19]	Long Dan Xie Gan granules: 6 g/po./tid	Long Dan Xie Gan granules: (same as Jiang F 2009)
	Ruyi Jinhuang paste (external): q.s./p.a.a./bid. or tid	Ruyi Jinhuang paste (external): (same as Jiang F 2009)

Yu F, 2013 [20]	Tanreqing injection: 20 ml/5% GS 250 ml ivgtt./qd	Tanreqing injection: *Scutellariae Radix* (huangqin), *Pulvis Fellis Ursi* (xiongdanfen), capra hircus cornu (shanyangjiao), *Lonicerae Japonicae Flos* (jinyinhua), *Fructus Forsythiae* (lianqiao), etc. Excipients: propylene glycol
	CHM wash lotion: 1,000∼2,000 mL/p.a.a. for 20∼25 min/bid	CM wash lotion: *Sophorae Flavescentis Radix* (kushen), *Rehmanniae Radix* (shengdi), *Phellodendri Chinensis Cortex* (huangbo), *Corydalis Rhizoma* (yuanhu, duanmuli, wubeizi), *Lonicerae Japonicae Flos* (jinyinhua, tufuling), *Taraxaci Herba* (pugongying), *Alumen* (baifan), *Alismatis Rhizoma* (zexie), and *Borneolum* (bingpian)

Duan XW, 2011 [21]	Long Dan Xie Gan granules: unknown/po./bid	Long Dan Xie Gan granules: *Gentianae Radix et Rhizoma* (longdan), *Gardeniae Fructus* (zhizi), *Scutellariae Radix* (huangqin), *Bupleuri Radix* (chaihu), *Rehmanniae Radix* (shengdi), *Plant Aginis Semen* (cheqianzi-with hot salt frying), *Alismatis Rhizoma* (zexie), *Tetrapanacis Medulla* (tongcao), *Glycyrrhizae Radix et Rhizoma* (gancao), *Paeoniae Radix Rubra* (chishao), *Toosendan Fructus* (chuanlianzi), *Artemisiae Scopariae Herba* (yinchen), and *Lonicerae Japonicae Flos* (jinyinhua)
Zeng L, 2005 [22]	Long Dan Xie Gan granules: elixation/po./bid	Long Dan Xie Gan granules: unknown
	Jidesheng sheyao tablets (external): grind into powder and mix with water to make paste, q.s./p.a.a./q3h	Jidesheng sheyao tablets (external): *Paridis Rhizoma* (chonglou), *Scolopendra* (wugong), *Euphorbiae Humifusae Herba* (dijincao), etc.

Liang FL, 2012 [23]	Herbal gargle: add 500 mL water boil to 250 mL elixation/p.a.a. 20∼25 min/bid (patients with oral herpes) 30–50 ml/Garg.3∼5 min/bid	Herbal gargle: *Sophorae Flavescentis Radix* (kushen), *Rehmanniae Radix* (shengdi), *Scrophulariae Radix* (xuanshen), *Ophiopogonis Radix* (maidong), *Adenophorae Radix* or *Glehniae Radix* (shashen), *Gynostemma Pentaphyllum* (jiaogulan), *Ilexasprella Radix* (gangmeigen), and *Coptidis Rhizoma* (huanglian)
Wang Q, 2014 [24]	Long Dan Xie Gan formula or Ba Zhen formula: elixation/po./bid	Long Dan Xie Gan formula: *Gentianae Radix et Rhizoma* (longdan), *Gardeniae Fructus* (zhizi), *Scutellariae Radix* (huangqin), *Bupleuri Radix* (chaihu), *Rehmanniae Radix* (shengdi), *Plant Aginis Semen* (cheqianzi-with hot salt frying), *Alismatis Rhizoma* (zexie), *Tetrapanacis Medulla* (tongcao), *Polygoni Cuspidate Rhizome et Radix* (huzhang), *Arneblae Radix* (zicao), and *Lonicerae Japonicae Flos* (jinyinhua)
		Ba Zhen formula: *Rehmanniae Radix Praeparata* (shudi), *Codonopsis Radix* (dangshen), *Atractylodis Macrocephalae Rhizoma* (jiaobaizhu), *Angelicae Sinensis Radix* (dangui), *Chuanxiong Rhizoma* (chuanxiong), *Paeoniae Radix Alba* (chaobaishao), *Saposhnikoviae Radix* (fangfeng), *Poria* (fuling), *Glycyrrhizae Radix et Rhizoma Praeparata Cum Melle* (zhigancao), *Polygoni Cuspidate Rhizome et Radix* (huzhang), *Arneblae Radix* (zicao), and *Lonicerae Japonicae Flos* (jinyinhua)

Shao Z, 2011 [25]	Long Dan Xie Gan formula or chushi	Longda nxiegan formula: (Same as duan XW 2011)
	Weiling formula: elixation half dose/po./bid	Chu Shi Wei Ling formula: *Atractylodis Rhizoma* (cangzhu), *Atractylodis Macrocephalae Rhizoma* (baizhu), *Toosendan Fructus* (chuanlianzi), *Citri Reticulatae Pericarpium* (chenpi), *Polyporus* (zhuling), *Poria* (fuling), *Alismatis Rhizoma* (zexie), *Tetrapanacis Medulla* (tongcao), *Coicis Semen* (yiyiren), *Astragali Radix* (huangqi), *Codonopsis Radix* (dangshen), *Corydalis Rhizoma* (yuanhu), *Scolopendra* (wugong), and *Glycyrrhizae Radix et Rhizoma* (gancao)
	Sanhuang wash lotion: q.s./po./unknown	Sanhuang wash lotion: *Phellodendri Chinensis Cortex* (huangbo), *Portulacae Herba* (machixian), indigo naturalis (qingdai), excipients: sesame oil

Pan HR, 2010 [26]	Long Dan Xie Gan formula and chushi	Long Dan Xie Gan formula: (same as Duan XW, 2011, Shao Z, 2011)
	Weiling formula: elixation/po./bid	Chu Shi Wei Ling formula: (same as Shao Z, 2011)
	Sanhuang wash lotion: q.s./po./unknown	Sanhuang wash lotion: (same as Shao Z, 2011)

Wu JL, 2011 [27]	Long Dan Xie Gan formula or chushi weiling formula: elixation/unknown/unknown	Longda nxiegan formula: unknown
		Chu Shi Wei Ling formula: unknown
	Method/specifications/retention time	**Operating site/acupoint selection** ^*∗*^

Liu ZW, 2013 [28]	Encircling needling/0.38 mm*∗*25 mm, 15°/20 min thread-moxa/0.7 mm*∗*300 mm/sparkle press acupoint for 2∼3 times	Acupuncture: surround needling for herpes periphery/no special acupoint
	Jingwanhong burn ointment: q.s./po./after thread-moxa	Thread-moxa: for small herpes clusters/main acupoint: ashi points; matching points: zusanli (st36, bilateral) and guanyuan (rn4). after thread-moxa apply jingwanhong burn ointment on herpes, for big herpes prick before applying. composition of jingwanhong burn ointment: unknown

Li M, 2000 [29]	Regular moxibustion: unknown/31 min	Moxibustion: herpes area/no special acupoint
	Blood-letting puncture: three-edged needle/several drops of blood	Blood-letting puncture: healthy skin around herpes/no special acupoint
	Reducing acupuncture method: 0.38 mm*∗*25 mm, 15°/30 min	Reducing acupuncture method: determined acupoint/fengchi (GB20), quchi (LI11), hegu (LI4), taichong (LR3), zusanli (ST36), yinlingquan (SP9), and sanyinjiao (SP6)
	Encircling needling + reducing acupuncture method: 0.38 mm*∗*25 mm, 15°/30 min	Encircling needling: healthy skin around herpes/needling around herpes, each needle interval for 5 cm/no special acupoint
	Transverse needling: 0.38 mm*∗*25 mm, 15°/30 min	Transverse needling: inside herpes range/needling on herpes lesion area, each needle interval for 5 cm side by side/no special acupoint

^*∗*^Details of acupuncture and moxibusion are provided in Additional file 2.

**Table 3:** Summary report of different outcomes and effect estimates.

Comparison group	Outcomes	Study ID	Sample size I/C (events)	Effect estimation [95% CI]	*P* (*α* = 0.05)
CHM vs. drugs	Pain score (VAS)	Total	45/48	MD −0.87 [−1.69, −0.04]	0.04
		Jiang F, 2009	30/30	MD −1.04 [−2.05, −0.03]	0.04
		Meng L, 2006	15/18	MD −0.52 [−1.96, 0.92]	0.48
	**Cure rate (end of treatment)**	**Total**	**45/48 (35/23)**	**RR 1.58 [1.13, 2.22]**	**0.008**
		Meng L, 2006	15/18 (11/9)	RR 1.38 [0.78, 2.43]	0.18
		Jiang F, 2009	30/30 (24/14)	RR 1.71 [1.12, 2.62]	0.01

CHM + drugs vs. drugs	**Pain score (VAS)**	Shao Z, 2011	18/16	MD −0.47 [−1.38, 0.44]	0.31
	**Duration of pain (days)**	**Total**	**73/71**	**MD** −**9.19** [−**16.73,** −**1.65]**	**0.02**
		Duan XW, 2011	10/10	MD −16.00 [−23.32, −8.68]	<0.0001
		Wang Q, 2014	35/35	MD −1.70 [−2.80, −0.60]	0.003
		Pan HR, 2010	10/10	MD −16.00 [−23.32, −8.68]	<0.0001
		Shao Z, 2011	18/16	MD −5.46 [−10.89, −0.03]	0.05
	Incidence of PHN	**Total**	**101/101 (10/21)**	**RR 0.49 [0.25, 0.99]**	**0.05**
		Liang FL, 2012	30/30 (3/9)	RR 0.33 [0.10, 1.11]	0.07
		Yu F, 2013	36/36 (6/9)	RR 0.67 [0.26, 1.68]	0.39
		Wang Q, 2014	35/35 (1/3)	RR 0.33 [0.04, 3.05]	0.33
	**Cure rate (end of treatment)**	**Total**	**112/112 (62/44)**	**RR 1.40 [1.08, 1.82]**	**0.01**
		Duan XW, 2011	10/10 (10/6)	**RR** 1.62 [0.97, 2.69]	0.06
		Zeng L, 2005	18/20 (10/8)	**RR** 1.39 [0.71, 2.73]	0.34
		Liang FL, 2012	30/30 (10/8)	**RR** 1.25 [0.57, 2.73]	0.57
		Shao Z, 2011	18/16 (11/4)	**RR** 2.44 [0.97, 6.17]	0.06
		Yu F, 2013	36/36 (21/18)	**RR** 1.17 [0.76, 1.79]	0.48
	**Quality of life (WHOQOL-100)**	**Total**	**94/57**	**MD 4.72 [0.45, 8.98]**	**0.03**
		Shao Z, 2011	18/16	MD 7.59 [1.06, 14.12]	0.02
		Wu JL, 2011	76/41	MD 3.07 [−1.55, 7.69]	0.19
AM vs. drugs	**Pain score (VAS)**	Liu ZW, 2013	30/30	MD −1.10 [−1.70, −0.50]	0.0003
	**Duration of pain (days)**	Liu ZW, 2013	30/30	MD −5.87 [−7.85, −3.89]	<0.00001
	**Cure rate (end of treatment)**	**Total**	**70/50 (46/16)**	**RR 1.99 [1.18, 3.36]**	**0.01**
		Liu ZW, 2013	30/30 (18/11)	**RR** 1.64 [0.94, 2.85]	0.08
		Li M, 2000	40/20 (28/5)	**RR** 2.80 [1.28, 6.14]	0.01

Note: CHM – Chinese herbal medicine, AM – acupuncture and moxibustion, MD – mean difference, CI – confidence interval, RR – risk radio, and PHN – postherpetic neuralgia.

## Data Availability

No data were used to support this study.

## Abbreviations

HZ: Herpes zoster
HIV: Human immunodeficiency virus
CNKI: China national knowledge infrastructure
TCM: Traditional Chinese medicine
CHM: Chinese herbal medicine
PHN: Postherpetic neuralgia
RCTs: Randomized controlled trials
CI: Confidence interval
QOL: Quality of life
RR: Risk ratio
MD: Mean difference
I: Intervention group
C: Control group
M: Male
F: Female

## Supplementary Materials

Supplementary Materials Additional file 1: Searching strategies. Additional file 2: The details of intervention for included study. Additional file 3: The percentage figure for risk of bias. Additional file 4-1: The details of the risk of bias assessment-Domain 1. Randomization process. Additional file 4-2: The details of the risk of bias assessment-Domain 2. Deviations from intended interventions. Additional file 4-3: The details of the risk of bias assessment-Domain 3. Missing outcome data. Additional file 4-4: The details of the risk of bias assessment-Domain 4. Measurement of the outcome. Additional file 4-5: The details of the risk of bias assessment-Domain 5. Selection of the reported result.
