# Supplementary material for: Traditional Chinese Medicine for HIV-Associated Acute Herpes Zoster: A Systematic Review and Meta-Analysis of Randomized Trials
Source: Evid Based Complement Alternat Med. 2022 Feb 18;2022:8674648. doi: 10.1155/2022/8674648 (PMC8881158; doi:10.1155/2022/8674648)
Supplement: Supplementary Materials — Additional file 1: Searching strategies. Additional file 2: The details of intervention for included study. Additional file 3: The percentage figure for risk of bias. Additional file 4-1: The details of the risk of bias assessment-Domain 1. Randomization process. Additional file 4-2: The details of the risk of bias assessment-Domain 2. Deviations from intended interventions. Additional file 4-3: The details of the risk of bias assessment-Domain 3. Missing outcome data. Additional file 4-4: The details of the risk of bias assessment-Domain 4. Measurement of the outcome. Additional file 4-5: The details of the risk of bias assessment-Domain 5. Selection of the reported result. [file 8674648.f1.docx]

**Additional file-1-Searching strategies**

| Searching Terms ： | acquired immune deficiency syndrome, acquired immuno-deficiency syndrome, human immunodeficiency virus, human immune deficiency virus, hiv, aids, Chinese herb, Chinese medicine, traditional medicine, alternative medicine, complementary medicine, complementary therapy, herpes zoster, shingles, neuralgia, postherpetic, zoster, postherpetic neuralgia, post?herpetic neuralgia | |
| --- | --- | --- |
| Database | Last search time  Citations | Search terms |
| China National Knowledge Infrastructure (CNKI) | 1994 - 5^th^ 12 2021  99 citations | SU %= (Chinese herbal medicine + Chinese medicine + complementary medicine + traditional medicine + herbal medicine + ethnodrug + folk recipe + prepared herbal medicine + ingredient + pill + pulvis + medicinal extract plaster + needle + moxibustion + acupoint + cupping + massage + Tuina + scrapping therapy) AND SU %= (acquired immune deficiency syndrome + AIDS + human immune deficiency virus + HIV) AND SU %=(random + randomized control trail + cohort study + case-control study) AND FT=(herpes zoster + shingles + postherpetic neuralgia) NOT TI=(animal + rats + dog + rabbit + monkey) |
| Wanfang Database | 29 Jan 2020 - 5^th^ 12 2021  55 citations | SU:(Chinese herbal medicine + Chinese medicine + complementary medicine + traditional medicine + herbal medicine + ethnodrug + folk recipe + prepared herbal medicine + ingredient + pill + pulvis + medicinal extract plaster + needle + moxibustion + acupoint + cupping + massage + Tuina + scrapping therapy)*SU:(acquired immune deficiency syndrome + AIDS + human immune deficiency virus + HIV)*SU:(random + randomized control trail + cohort study + case-control study)*SU:(herpes zoster + shingles + postherpetic neuralgia)^ TI:(animal + rats + dog + rabbit + monkey) |
| Chongqing VIP | 29 Jan 2020 - 5^th^ 12 2021  11 citations | (R=Chinese herbal medicine + Chinese medicine + complementary medicine + traditional medicine + herbal medicine + ethnodrug + folk recipe + prepared herbal medicine + ingredient + pill + pulvis + medicinal extract plaster + needle + moxibustion + acupoint + cupping + massage + Tuina + scrapping therapy) AND (R=acquired immune deficiency syndrome + AIDS + human immune deficiency virus + HIV) AND (R=random + randomized control trail + cohort study + case-control study) NOT (M=animal + rats + dog + rabbit + monkey) |
| SinoMed | 29 Jan 2020 - 5^th^ 12 2021  58 citations | (((((((((((((((((((((("Chinese herbal medicine"[Common field]) OR "Chinese medicine"[Common field]) OR " complementary medicine"[Common field]) OR "prepared herbal medicine"[Common field]) OR "herbal medicine"[Common field]) OR "ingredient"[Common field]) OR "pill"[Common field]) OR "pulvis"[Common field]) OR "plaster"[Common field]) OR "ethnodrug"[Common field]) OR "folk recipe"[Common field]) OR "needle"[Common field]) OR "moxibustion"[Common field]) OR "acupoint"[Common field]) OR "cupping"[Common field]) OR "massage"[Common field]) OR "Tuina"[Common field]) OR "scrapping therapy"[Common field]  ((((("acquired immune deficiency syndrome"[Common field]) OR "HIV"[Common field]) OR "human immune deficiency virus"[Common field]) OR "AIDS"[Common field])  ("herpes zoster"[All text] OR "shingles"[All text] OR "postherpetic neuralgia"[All text]) |
| ClinicalTrials.gov | Since establish - 5^th^ 12 2021  1 citation | #1 Title: Herbal medicines for treating HIV infection and AIDS  Recruitment Status: All Studies  Study Results: All Studies  Study Type: All Studies  Gender: All Studies  #2 Condition: HIV or ADIS or human immune deficiency virus or human immuno deficiency virus or human immunodeficiency virus or acquired immunodeficiency syndrome or acquired immune deficiency syndrome or viral sexually transmitted diseases  Intervention: Traditional Chinese Medicine OR TCM OR Chinese herbal medicine OR Chinese medicinal herb OR herbal medicine OR medicinal plant OR Chinese herbal drug OR Chinese herbal formula OR Chinese herb drug  Recruitment Status: All Studies  Study Results: All Studies  Study Type: All Studies  Gender: All Studies  #3 Condition: HIV or ADIS or human immune deficiency virus or human immuno deficiency virus or human immunodeficiency virus or acquired immunodeficiency syndrome or acquired immune deficiency syndrome or viral sexually transmitted diseases  Intervention: herb AND (extract or single OR drug or compound or mixture)  Recruitment Status: All Studies  Study Results: All Studies  Study Type: All Studies  Gender: All Studies  #4 #1 or #2 or #3 |
| WHO International Clinical Trial Registration Platform (WHO ICTRP) | Since establish - 5^th^ 12 2021  33 citations | #1 Title: Herbal medicines for treating HIV infection and AIDS, Recruitment Status: ALL  #2 Condition: HIV or ADIS or human immune deficiency virus or human immuno deficiency virus or human immunodeficiency virus or acquired immunodeficiency syndrome or acquired immune deficiency syndrome or viral sexually transmitted diseases, Intervention: Traditional Chinese Medicine OR TCM OR Chinese herbal medicine OR Chinese medicinal herb OR herbal medicine OR medicinal plant OR Chinese herbal drug OR Chinese herbal formula OR Chinese herb drug, Recruitment Status: ALL  #3 Condition: HIV or ADIS or human immune deficiency virus or human immuno deficiency virus or human immunodeficiency virus or acquired immunodeficiency syndrome or acquired immune deficiency syndrome or viral sexually transmitted diseases, Intervention: herb AND (extract or single OR drug or compound or mixture), Recruitment Status: ALL  #4 #1 or #2 or #3 |
| Cochrane Central Register of Controlled Trials (CENTRAL) on The Cochrane Library | Since establish - 5^th^ 12 2021  61 citations  (12 trials, 45 SRs, 3 Protocols, 1 clinical answers) | #1 MeSH descriptor: [HIV Infections] explode all trees  #2 MeSH descriptor: [HIV] explode all trees  #3 MeSH descriptor: [Acquired Immunodeficiency Syndrome] explode all trees  #4 MeSH descriptor: [Lymphoma, AIDS-Related] this term only  #5 MeSH descriptor: [Sexually Transmitted Diseases, Viral] this term only  #6 ('HIV infect*' or 'hiv1' or 'hiv2' or 'hiv-1*' or 'hiv-2*' or HIV or 'human immunodeficiency virus' or 'human immune-deficiency virus' or 'human immune deficiency virus' or 'human immuno deficiency virus' or 'human immun*' or 'deficiency virus' or 'acquired immunodeficiency syndrome' or 'acquired immune-deficiency syndrome' or 'acquired immune deficiency syndrome' or ' acquired immuno deficiency syndrome ' or 'acquired immun*' or 'deficiency syndrome' or 'viral sexually transmitted diseases'):ti,ab,kw (Word variations have been searched)  #7 {OR #1-#6}  #8 MeSH descriptor: [Drugs, Chinese Herbal] explode all trees  #9 MeSH descriptor: [Phytotherapy] explode all trees  #10 MeSH descriptor: [Herbal Medicine] explode all trees  #11 MeSH descriptor: [Plants, Medicinal] explode all trees  #12 MeSH descriptor: [Plant Preparations] explode all trees  #13 MeSH descriptor: [Plant Extracts] explode all trees  #14 MeSH descriptor: [Medicine, Kampo] explode all trees  #15 MeSH descriptor: [Medicine, East Asian Traditional] explode all trees  #16 ((herbal near/6 remed*) or (herbal near/6 extract*) or (herbal near/6 preparation*) or (herbal near/6 mixture*) or (herbal near/6 medic*))  #17 ((phyto near/6 drug*) or (phyto near/6 pharmaceutical*) or (phyto near/6 therap*) or (phyto near/6 treatment*) or (phyto near/6 medici*))  #18 ((Chinese near/6 herb*) or (Chinese near/6 plant*) or (Chinese near/6 medic*) or (Chinese near/6 drug*) or (Chinese near/6 formul*) or (Chinese near/6 prescri*))  #19 ((plant* near/6 preparation*) or (plant* near/6 extract*) or (plant* near/6 medic*))  #20 ((Chinese near/6 traditional) and medic*)  #21 (complementary near/3 therap*) or (complementary near/3 medicin*) or (complementary near/3 treatment*)  #22 (alternativ* near/3 therap*) or (alternativ* near/3 medicin*)  #23 (botanical and extract*)  #24 MeSH descriptor: [Acupuncture] explode all trees  #25 MeSH descriptor: [Acupuncture Therapy] explode all trees  #26 acupunct* or acupress* or acupoint* or electroacupunct* or auriculotherap* or auriculoacupunct* or moxibust* or meridian* or patch*  #27 cupping therapy or Chinese massage or Tuina or skin scraping therapy  #28 {OR #8-#27}  #29 MeSH descriptor: [Herpes Zoster] explode all trees  #30 MeSH descriptor: [Neuralgia, Postherpetic] explode all trees  #31 (herpes zoster or shingles or zoster or postherpetic neuralgia or post-herpetic neuralgia or post herpetic neuralgia) (Word variations have been searched)  #32 {OR #29-#31} 2988  #33 #7 AND #28 AND #32 |
| PubMed | Since establish - 5^th^ 12 2021  54 citations | #1 HIV Infections[MeSH] OR HIV[MeSH] OR hiv[tw] OR hiv-1*[tw] OR hiv-2*[tw] OR hiv1[tw] OR hiv2[tw] OR hiv infect*[tw] OR human immunodeficiency virus[tw] OR human immune deficiency virus[tw] OR human immuno-deficiency virus[tw] OR human immune-deficiency virus[tw] OR ((human immun*) AND (deficiency virus[tw])) OR acquired immunodeficiency syndrome[tw] OR acquired immune deficiency syndrome[tw] OR acquired immuno-deficiency syndrome[tw] OR acquired immune-deficiency syndrome[tw] OR ((acquired immun*) AND (deficiency syndrome[tw])) OR "sexually transmitted diseases, viral"[MESH:NoExp]  #2 medicine, traditional[MeSH] OR medicine, chinese traditional[MeSH] OR Complementary Therapies[MeSH] OR plant extracts[MeSH] OR drugs, chinese herbal[MeSH] OR Herbal Medicine[MeSH] OR Plants, Medicinal[MeSH] OR Plant preparations[MeSH] OR Medicine, Kampo[MeSH] OR Phytotherapy[MeSH] OR Acupuncture[MeSH] OR Acupuncture Therapy[MeSH] OR (Chinese medicin*[tiab]) OR (Chinese herb*[tiab]) OR (Chinese plant*[tiab]) OR (Chinese drug*[tiab]) OR (Chinese formul*[tiab]) OR (Chinese prescri*[tiab]) OR (oriental medicin*[tiab]) OR (traditional medicin*[tiab]) OR (alternativ* medicin*[tiab]) OR (alternativ* therap*[tiab]) OR (complementary medicin*[tiab]) OR (complementary therap*[tiab]) OR (complementary treatment*[tiab]) OR (botanical extract*[tiab]) OR (plant extract*[tiab]) OR (plant preparation*[tiab]) OR (plant medicin*[tiab]) OR herb*[tiab] OR (herbal remed*[tiab]) OR (herbal extract*[tiab]) OR (herbal preparation*[tiab]) OR (herbal mixture*[tiab]) OR (herbal medicin*[tiab]) OR (phyto drug*[tiab]) OR (phyto pharmaceutical*[tiab]) OR (phyto therap*[tiab]) OR (phyto treatment*[tiab]) OR (phyto medicin*[tiab]) OR (phytopharmaceutic*[tiab]) OR (acupunct*[tiab]) OR (acupress*[tiab]) OR (acupoint*[tiab]) OR (electroacupunct*[tiab]) OR (auriculotherap*[tiab]) OR (auriculoacupunct*[tiab]) OR (moxibust*[tiab]) OR (meridian*[tiab]) OR (patch*[tiab]) OR (cupping therapy[tiab]) OR (Chinese massage[tiab]) OR (Tuina[tiab]) OR (skin scraping therapy[tiab])  #3 (randomized controlled trial [pt] OR controlled clinical trial [pt] OR randomized [tiab] OR placebo [tiab] OR drug therapy [sh] OR randomly [tiab] OR trial [tiab] OR groups [tiab] OR crossover or cross-over [tw] OR pragmatic clinical trial [pt] OR cohort [pt] OR case-control [pt] OR case series) NOT (animals [mh] NOT humans [mh])  #4 Clinical Trials as Topic[MeSH Terms]  #5 Pragmatic Clinical Trials as Topic[MeSH Terms]  #6 Meta-Analysis as Topic[MeSH Terms]  #7 [Controlled Clinical Trial](https://www.ncbi.nlm.nih.gov/pubmed)  #8 [Observational Study](https://www.ncbi.nlm.nih.gov/pubmed)  #9 [Randomized Controlled Trial](https://www.ncbi.nlm.nih.gov/pubmed)  #10 [Systematic Reviews](https://www.ncbi.nlm.nih.gov/pubmed)  #11 #3 OR #4 OR #5 OR #6 OR #7 OR #8 OR #9 OR #10  #12 (((((((herpes zoster[MeSH Terms]) OR (shingles[MeSH Terms])) OR (neuralgia, postherpetic[MeSH Terms])) OR (herpes zoster)) OR (shingles)) OR (zoster)) OR (postherpetic neuralgia)) OR (post?herpetic neuralgia)  #13 #1 AND #2 AND #11 AND #12 |
| EMBASE via OVID | Since establish - 5^th^ 12 2021  13 citations | #1 (‘human immunodeficiency virus infection’/exp OR ‘human immunodeficiency virus infection’) OR ((‘human immunodeficiency virus’/exp OR ‘human immunodeficiency virus')) OR (('b cell lymphoma'/de OR 'b cell lymphoma')) OR (hiv:ti OR hiv:ab) OR ('hiv-1':ti OR 'hiv-1':ab) OR ('hiv-2':ti OR 'hiv-2':ab) OR ('human immunodeficiency virus':ti OR 'human immunodeficiency virus':ab) OR ('human immune deficiency virus':ti OR 'human immune deficiency virus':ab) OR ('human immune-deficiency virus':ti OR 'human immune-deficiency virus':ab) OR ('human immuno-deficiency virus':ti OR 'human immuno-deficiency virus':ab) OR ('acquired immunodeficiency syndrome':ti OR 'acquired immunodeficiency syndrome':ab) OR ('acquired immuno-deficiency syndrome':ti OR 'acquired immuno-deficiency syndrome':ab) OR ('acquired immune-deficiency syndrome':ti OR 'acquired immune-deficiency syndrome':ab) OR ('acquired immune deficiency syndrome':ti OR 'acquired immune deficiency syndrome':ab)  #2 'alternative medicine'/exp OR 'traditional medicine'/exp OR 'chinese medicine'/exp OR 'Plant Extract'/exp OR 'Chinese Drug'/exp OR 'Chinese Herb'/exp OR 'Medicinal Plant'/exp OR 'Kampo medicine'/exp OR 'Herb'/exp OR 'Chinese medicin*':ti,ab OR 'Chinese herb*':ti,ab OR 'Chinese plant*':ti,ab OR 'Chinese drug*':ti,ab OR 'Chinese formul*':ti,ab OR 'Chinese prescri*':ti,ab OR 'oriental medicin*':ti,ab OR 'traditional medicin*':ti,ab OR 'alternativ* medicin*':ti,ab OR 'alternativ* therap*':ti,ab OR 'complementary medicin*':ti,ab OR 'complementary therap*':ti,ab OR 'complementary treatment*':ti,ab OR 'botanical extract*':ti,ab OR 'plant* extract*':ti,ab OR 'plant* preparation*':ti,ab OR 'plant* medic*':ti,ab OR herb*:ti,ab OR 'herbal remed*':ti,ab OR 'herbal extract*':ti,ab OR 'herbal preparation*':ti,ab OR 'herbal mixture*':ti,ab OR 'herbal medicin*':ti,ab OR 'phyto drug*':ti,ab OR 'phyto pharmaceutical*':ti,ab OR 'phyto therap*':ti,ab OR 'phyto treatment*':ti,ab OR 'phyto medicin*':ti,ab OR phytopharmaceutic*:ti,ab OR 'acupunct*':ti,ab OR 'acupress*':ti,ab OR 'acupoint*':ti,ab OR 'electroacupunct*':ti,ab OR 'auriculotherap*':ti,ab OR 'auriculoacupunct*':ti,ab OR 'moxibust*':ti,ab OR 'meridian*':ti,ab OR 'patch*':ti,ab OR 'cupping therapy':ti,ab OR 'Chinese massage':ti,ab OR 'Tuina':ti,ab OR 'skin scraping therapy':ti,ab  #3 (random*:ti OR random*:ab) OR (open adj label:ti OR open adj label:ab) OR (factorial*:ti OR factorial*:ab) OR (compare or compared or comparison:ti) OR (cross?over*:ti OR cross?over*:ab OR crossover*:ti OR crossover*:ab) OR (placebo*:ti OR placebo*:ab) OR ((doubl*:ti AND blind*:ti) OR (doubl*:ab AND blind*:ab)) OR ((singl*:ti AND blind*:ti) OR (singl*:ab AND blind*:ab)) OR (assign*:ti OR assign*:ab) OR (allocat*:ti OR allocat*:ab) OR (volunteer*:ti OR volunteer*:ab) OR (('crossover procedure'/de OR 'crossover procedure')) OR (('double blind procedure'/de OR 'double blind procedure')) OR (('single-blind procedure'/de OR 'single-blind procedure')) OR (('randomized controlled trial'/de OR 'randomized controlled trial')) OR (parallel group$1:ti OR parallel group$1:ab) OR ((controlled adj7 (study or design or trial)):ti OR (controlled adj7 (study or design or trial)):ab) OR (((assign$ or match or matched or allocation) adj5 (alternate or group$1 or intervention$1 or patient$1 or subject$1 or participant$1)):ti OR ((assign$ or match or matched or allocation) adj5 (alternate or group$1 or intervention$1 or patient$1 or subject$1 or participant$1)):ab) OR (cohort:ti OR cohort:ab) OR (case-control:ti OR case-control:ab) OR (case series:ti OR case series:ab)  #4 ALL=('herpes zoster'/exp OR zoster OR shingles OR 'neuralgia, postherpetic'/exp OR postherpetic neuralgia OR post-herpetic neuralgia OR post herpetic neuralgia)  #5 #1 AND #2 AND #3 AND #4 |

SU：Topic；FT：Full text；TI：Title；TCM：Traditional Chinese Medicine; CM: Chinese Medicine

**Additional file 2 The details of Intervention for included study**

| Study ID | Intervention specifications | Medicine: Doasge(for 1 use) /Usage /Frequenc | Course of treatment | Composition of CHM |
| --- | --- | --- | --- | --- |
| CHM vs. Drugs | | | | |
| Jiang F 2009 | I: Longdan Xiegan granules + Ruyi Jinhuang Paste (external)  C: Acyclovir | Longdan Xiegan granules: 6g /po. /tid.;  Ruyi Jinhuang Paste (external): q.s. /p.a.a. /bid. or tid.  Acyclovir: 0.2g /po. /5times per day;  Acyclovir ointment(external): q.s. /p.a.a. /bid. or tid. | 10days/course, 1 course | Longdan Xiegan granules : Gentianae Radix et Rhizoma (Longdan), Gardeniae Fructus (Zhizi), Scutellariae Radix (Huangqin), Bupleuri Radix (Chaihu), Rehmanniae Radix (Shengdi), Plant Aginis Semen (Cheqianzi-with Hot salt frying), Alismatis Rhizoma (Zexie), Aristolochia Manshuriensis (Guanmutong), Angelicae Sinensis Radix (Danggui-with Hot alcohol frying), Glycyrrhizae Radix et Rhizoma Praeparata Cum Melle (Zhigancao).  Ruyi Jinhuang Paste (external): Trichosanthis Radix (Tianhuafen), Curcumae Longae Rhizoma (Jianghuang), Paeoniae Radix Alba (Baizhi), Atractylodis Rhizoma (Cangzhu), Arisaemants Rhizoma (Tiannanxing), Glycyrrhizae Radix et Rhizoma (Gancao), Rhei Radix et Rhizoma (Dahuang), Phellodendri Chinensis Cortex (Huangbo), Magnoliae Officinalis Cortex (Houpo), Citri Reticulatae Pericarpium (Chenpi), Excipients: Sesame oil, lead tetroxide. |
| Meng L 2006 | I: Longdan Xiegan granules + Ruyi Jinhuang Paste (external)  C: Acyclovir | Longdan Xiegan granules: 6g /po. /tid.;  Ruyi Jinhuang Paste (external): q.s. /p.a.a. /bid. or tid.  Acyclovir: 0.2g /po. /5times per day;  Acyclovir ointment(external): q.s. /p.a.a. /bid. or tid. | 14days/course, 1 course | Longdan Xiegan granules : （Same as Jiang F 2009）  Ruyi Jinhuang Paste (external): （Same as Jiang F 2009） |
| CHM + Drugs vs. Drugs | | | | |
| Yu F 2013 | I: Tanreqing injection + CHM wash lotion + Acyclovir + Diclofenac sodium + Carbamazepine  C: Acyclovir + Diclofenac sodium + Carbamazepine | Tanreqing injection: 20ml /5% GS 250ml ivgtt. /qd..  CHM wash lotion: 1000～2000mL / p.a.a. for 20～25min /bid.  Acyclovir: 5~10(mg/kg) /5% GS 250ml vgtt. /q8h.  Acyclovir ointment(external): q.s. /p.a.a. /Unknown  Diclofenac sodium: Discretion /Unknown /Unknown  Carbamazepine: Discretion /Unknown /Unknown | 7days/course, 3 course | Tanreqing injection: Scutellariae Radix (Huangqin), Pulvis Fellis Ursi (Xiongdanfen), Capra Hircus Cornu (Shanyangjiao), Lonicerae Japonicae Flos (Jinyinhua), Fructus Forsythiae (Lianqiao), etc. Excipients: Propylene Glycol.  CHM wash lotion : Sophorae Flavescentis Radix (Kushen), Rehmanniae Radix (Shengdi), Phellodendri Chinensis Cortex (Huangbo), Corydalis Rhizoma (Yuanhu), (Duanmuli), (Wubeizi), Lonicerae Japonicae Flos (Jinyinhua), (Tufuling), Taraxaci Herba (Pugongying), Alumen (Baifan), Alismatis Rhizoma (Zexie), Borneolum (Bingpian). |
| Duan XW 2011 | I: Longdan Xiegan granules + Valacyclovir  C: Valacyclovir | Longdan Xiegan granules: Unknown /po. /bid.  Valacyclovir: 200m /po. /bid. | 45days/course, 1 course | Longdan Xiegan granules : Gentianae Radix et Rhizoma (Longdan), Gardeniae Fructus (Zhizi), Scutellariae Radix (Huangqin), Bupleuri Radix (Chaihu), Rehmanniae Radix (Shengdi), Plant Aginis Semen (cheqianzi-with Hot salt frying), Alismatis Rhizoma (Zexie), Tetrapanacis Medulla (Tongcao), Glycyrrhizae Radix et Rhizoma (Gancao), Paeoniae Radix Rubra (Chishao), Toosendan Fructus (Chuanlianzi), Artemisiae Scopariae Herba (Yinchen), Lonicerae Japonicae Flos (Jinyinhua). |
| Zeng L 2005 | I: Longdan Xiegan formula + Jidesheng Sheyao tablets (external) + Acyclovir+ Vitamin B1 + VitaminB12 + Somedon  C: Acyclovir+ Vitamin B1 + vitaminB12 + Somedon | Longdan Xiegan formula: elixation / po./ bid.  Jidesheng Sheyao tablets (external): Grind into powder and mix with water to make paste, q.s. /p.a.a./ q3h.  Acyclovir: 10mg/(kg) /ivgtt. /q8h  Vitamin B1: Unknown /po./ Unknown  VitaminB12: Unknown /po./ Unknown  Somedon: p.r.n. /po./ Unknown | 7days/course, 3 course | Longdan Xiegan formula: Unknown.  Jidesheng Sheyao tablets (external): Paridis Rhizoma (Chonglou), (Ganchanpi), Scolopendra (Wugong), Euphorbiae Humifusae Herba (Dijincao) and etc. |
| Liang FL 2012 | I: Acyclovir + ibuprofen or Tramadol + Herbal gargle  C: Acyclovir + ibuprofen or Tramadol | Herbal gargle: add 500mL water boil to 250 mL elixation / p.a.a. 20~25min/ bid.  (Patients with Oral Herpes) 30-50 ml/ Garg.3~5min/ bid.  Acyclovir: 5~10mg/kg /ivgtt. /q8h.  Ibuprofen: p.r.n. /po./ Unknown  Tramadol: p.r.n. /po./ Unknown | 10days/course, 1-3 course | Herbal gargle: Sophorae Flavescentis Radix (Kushen), Rehmanniae Radix (Shengdi), Scrophulariae Radix (Xuanshen), Ophiopogonis Radix (Maidong), Adenophorae Radix or Glehniae Radix (Shashen), Gynostemma Pentaphyllum (Jiaogulan), Ilexasprella Radix (Gangmeigen), Coptidis Rhizoma (Huanglian). |
| Wang Q 2014 | I: TBSD （Longdan Xiegan formula, Bazhen formula）+ Ganciclovir + Unknown AA for external use + Compound glycyrrhizin injection + BCG -PSN + vitaminB12 + Calamine  C: Ganciclovir + Unknown AA for external use + Compound glycyrrhizin injection + BCG -PSN + vitaminB12 + Calamine | Longdan Xiegan formula: add water boil to elixation /po. /bid.  Bazhen formula: add water boil to elixation /po. /bid.  Ganciclovir: 0.5g/ivgtt. /q8h.  Compound glycyrrhizin injection: 40mL /5% GS. UnknoDrugs /Qd. 7times.  Unknown AA for external use: q.s. /p.a.a./ Unknown  BCG -PSN: 1mg /im. /qod. for 4times.  vitaminB12: q.s. /po./ Unknown  Calamine: p.r.n. /p.a.a./ Unknown | 7days/course, 2-3 course | Longdan Xiegan formula: Gentianae Radix et Rhizoma (Longdan), Gardeniae Fructus (Zhizi), Scutellariae Radix (Huangqin), Bupleuri Radix (Chaihu), Rehmanniae Radix (Shengdi), Plant Aginis Semen (cheqianzi-with Hot salt frying), Alismatis Rhizoma (Zexie), Tetrapanacis Medulla (Tongcao), Polygoni Cuspidate Rhizome et Radix (Huzhang), Arneblae Radix (Zicao), Lonicerae Japonicae Flos (Jinyinhua).  Bazhen formula: Rehmanniae Radix Praeparata (Shudi), Codonopsis Radix (Dangshen), Atractylodis Macrocephalae Rhizoma (Jiaobaizhu), Angelicae Sinensis Radix (Dangui), Chuanxiong Rhizoma (Chuanxiong), Paeoniae Radix Alba (Chaobaishao), Saposhnikoviae Radix (Fangfeng), Poria (Fuling), Glycyrrhizae Radix et Rhizoma Praeparata Cum Melle (Zhigancao), Polygoni Cuspidate Rhizome et Radix (Huzhang), Arneblae Radix (Zicao), Lonicerae Japonicae Flos (Jinyinhua). |
| Shao Z 2011 | I: TBSD （Longdan Xiegan formula, Chushi Weiling formula）+ Sanhuang wash lotion + Valacyclovir + Acyclovir  C: Valacyclovir + Acyclovir | Longdan Xiegan formula:0.5dose /po. /bid.  Chushi Weiling formula: /po. /bid.  Sanhuang wash lotion: q.s. /po./ Unknown  Valacyclovir: 300mg /po./ bid  Acyclovir: 5-10mg/(kg) /Unknown/q8h. | 28days/course, 1 course | Longda nxiegan formula: （Same as Duan XW 2011）  Chushi Weiling formula: Atractylodis Rhizoma (Cangzhu), Atractylodis Macrocephalae Rhizoma (Baizhu), Toosendan Fructus (Chuanlianzi), Citri Reticulatae Pericarpium (Chenpi), Polyporus (Zhuling), Poria (Fuling), Alismatis Rhizoma (Zexie), Tetrapanacis Medulla (Tongcao), Coicis Semen (Yiyiren), Astragali Radix (Huangqi), Codonopsis Radix (Dangshen), Corydalis Rhizoma (Yuanhu), Scolopendra (Wugong), Glycyrrhizae Radix et Rhizoma (Gancao).  Sanhuang wash lotion: Phellodendri Chinensis Cortex (Huangbo), Portulacae Herba (Machixian), Indigo Naturalis (Qingdai), Excipients: Sesame oil. |
| Pan HR 2010 | I: TBSD （Longdan Xiegan formula, Chushi Weiling formula）+ Sanhuang wash lotion + Valacyclovir + Acyclovir  C: Valacyclovir + Acyclovir | Longdan Xiegan formula: /po. /bid.  Chushi Weiling formula: /po. /bid.  Sanhuang wash lotion: q.s. /po./ Unknown  Valacyclovir: 300mg/ po./ bid  Acyclovir: 5-10mg/(kg) /Unknown/q8h. | 28days/course, 1 course | Longda nxiegan formula: （Same as Duan XW 2011, Shao Z 2011）  Chushi Weiling formula: （Same as Shao Z 2011）  Sanhuang wash lotion: （Same as Shao Z 2011） |
| Wu JL 2011 | I: TBSD （Longdan Xiegan formula or Chushi Weiling formula） + Valacyclovir + Acyclovir  C: Valacyclovir + Acyclovir | Longdan Xiegan formula: elixation /Unknown /Unknown  Chushi Weiling formula:elixation /Unknown /Unknown  Valacyclovir: 300mg/ po./ bid  Acyclovir: 5-10mg/(kg) /Unknown/q8h. | 21days/course, 1 course | Longda nxiegan formula: Unknown  Chushi Weiling formula: Unknown |
| AM vs. Drugs | | method/ specifications/ retention time |  | Operating site /Acupoint selection/ Operation detail |
| Liu ZW 2013 | I: Acupuncture + Thread-Moxa in Zhuang Folk Medicine + Jingwanhong burn ointment  C: Nimesulide + Valacyclovir + Vitamin B1 + Ribavirin ointment(external) | encircling needling / 0.38mm*25mm, 15°/ 20min  Thread-Moxa/ 0.7mm*300mm /sparkle press acupoint for 2~3times  Jingwanhong burn ointment: q.s. /po./ After Thread-Moxa  Valacyclovir: 250mg/p.o/ tid.  Nimesulide: 0.1g/ p.o./ bid.  Vitamin B1: 20mg/ po./ tid.  Ribavirin ointment(external): q.s. /p.a.a../ qid. | 7days/course, 2 course | Acupuncture: For herpes periphery, about 2.5cm around the outer margin of herpes area/ 3 needles at the bigger butt, 3 needles at slender end, other needles around the herpes, each needle interval for 5cm/ No-special acupoint.  Thread-Moxa: For small herpes clusters/ Main acupoint: Ashi points, adge of herpes and with severe pain (pain-range less than 3cm circle, 3 Moxa points; range between 3 to 5cm circle, 6~8 Moxa points; range over 5cm circle, 10~16 points); matching points: Zusanli (bilateral) and Guanyuan./ Hold with two figers at one end of the Medicine thread, exposed the thread for 5mm, lit it on the alcohol lamp, shake off the flame, and wait until it forms into a bead spark, quickly press the spark to the selected acupoint, each acupoint press-off the spark for 2-3 times. Generally, Moxa points on the acupoint around lesion was arranged as lotus shape. After Thread-Moxa apply Jingwanhong burn ointment on herpes, for big herpes prick befor applying.  Composition of Jingwanhong burn ointment: Unknown. |
| Li M 2000 | I: Moxibustion + TBSD Acupuncture(Blood-letting puncture + reducing acupuncture method, Encircling needling + Reducing acupuncture method, Transverse needling)  C: Acyclovir | Regular moxibustion: unknown/ 31min  Blood-letting puncture: three-edged needle/ several drop of blood  reducing acupuncture method: 0.38mm*25mm, 15°/ 30min  Encircling needling + Reducing acupuncture method: 0.38mm*25mm, 15°/ 30min  Transverse needling: 0.38mm*25mm, 15°/ 30min  Acyclovir: 200mg/p.o/ tid.  Acyclovir(external): q.s. /p.a.a../ tid. | 10days/course, (take 7days for rest between crouses)  2 course | Regular moxibustion: Herpes area/ Unknown/ No-special acupoint  Blood-letting puncture: Healthy skin around herpes/ Prick every 3 ~ 5cm with three-pronged needle and let out several drops of blood/ No-special acupoint.  Reducing acupuncture method: Determined acupoint/ Unknown/ Fengchi, Quchi, Hegu, Taichong , Zusanli, Yinlingquan, Sanyinjiao acupoint.  Encircling needling: Healthy skin around herpes/Needling around herpes, each needle interval for 5cm / No-special acupoint.  Transverse needling: Inside herpes range /Needling on herpes lesion area, each needle interval for 5cm side by side / No-special acupoint. |

**Additional file 3 The percentage Figure for risk of bias**


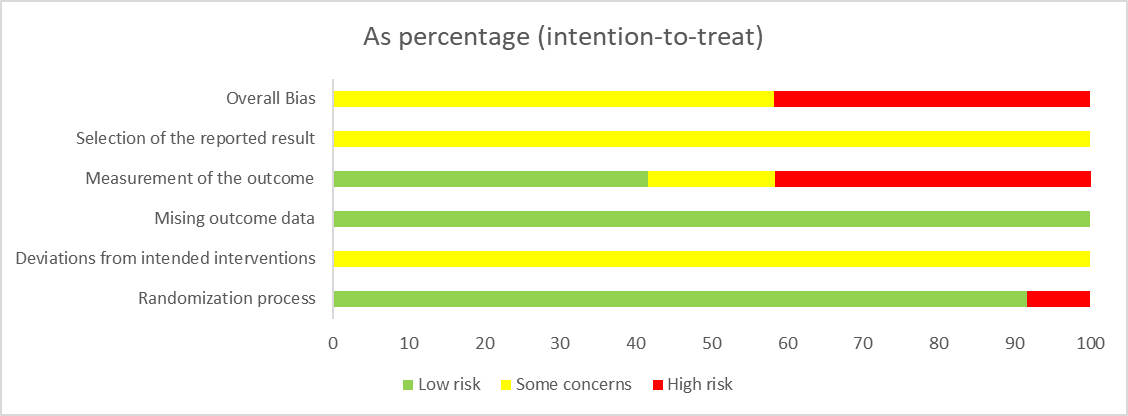


**Additional file 4-1 The details of risk of bias assessment- Domain 1. Randomization process**

| **Study ID** | **1.1** | **1.2** | **Note for 1.1&1.2** | **1.3** | **Note for 1.3** | **1.0 Assessor's Judgement** |
| --- | --- | --- | --- | --- | --- | --- |
| **Jiang F 2009** | PY | Y | Random  Sequentially numbered envelope  same team with Meng L 2006 and Liu ZW 2013 | N | comparable baseline  clear inclusion criteria and exclusion criteria | Low |
| **Meng L 2006** | Y | PY | Referring to a random number table same team with Jiang F 2009 and Liu ZW 2013 | PN |  | Low |
| **Yu F 2013** | NI | PY |  | N |  | Low |
| **Duan XW 2011** | Y | PY | random number table | N |  | Low |
| **Zeng L 2005** | NI | PY |  | PN |  | Low |
| **Liang FL 2012** | NI | PY |  | PN |  | Low |
| **Wang Q 2014** | NI | PY |  | PN |  | Low |
| **Shao Z 2011** | Y | Y | Using SPSS random number generator Central allocation system | N |  | Low |
| **Pan HR 2010** | PY | PY | same team with Shao Z 2011 | N |  | Low |
| **Wu JL 2011** | Y | Y | Central allocation (including telephone, web-based and pharmacy-controlled randomization) | N |  | Low |
| **Liu ZW 2013** | NI | PY | Alternate same team with Meng L 2006 and Liu ZW 2013 | PN |  | Low |
| **Li M 2000** | NI | PN |  | PN |  | High |

**Additional file 4-2 The details of risk of bias assessment- Domain 2. Deviations from intended interventions**

| **Study ID** | **2.1** | **2.2** | **Note for 2.1&2.2** | **2.3** | **Note for 2.3** | **2.4** | **2.5** | **Note for 2.5** | **2.6** | **Note for 2.6** | **2.7** | **Note for 2.7** | **2.0 Assessor's Judgement** |
| --- | --- | --- | --- | --- | --- | --- | --- | --- | --- | --- | --- | --- | --- |
| **Jiang F 2009** | PY | PY |  | NI | no protocol | NA | NA |  | Y |  | NA |  | Some concerns |
| **Meng L 2006** | PY | PY |  | NI | no protocol | NA | NA |  | NI |  | PN |  | Some concerns |
| **Yu F 2013** | PY | PY |  | NI | no protocol | NA | NA |  | NI |  | PN |  | Some concerns |
| **Duan XW 2011** | Y | PY | doctors are usually the one who performs the intervention | NI | no protocol | NA | NA |  | PY |  | NA |  | Some concerns |
| **Zeng L 2005** | PY | PY |  | NI | no protocol | NA | NA |  | PY |  | NA |  | Some concerns |
| **Liang FL 2012** | PY | PY |  | NI | no protocol | NA | NA |  | PY |  | NA |  | Some concerns |
| **Wang Q 2014** | PY | PY |  | NI | no protocol | NA | NA |  | PY |  | NA |  | Some concerns |
| **Shao Z 2011** | PY | PY |  | NI | no protocol | NA | NA |  | PY |  | NA |  | Some concerns |
| **Pan HR 2010** | PY | PY |  | NI | no protocol | NA | NA |  | NI |  | PN |  | Some concerns |
| **Wu JL 2011** | PY | PY |  | NI | no protocol | NA | NA |  | NI |  | PN |  | Some concerns |
| **Liu ZW 2013** | Y | Y |  | NI | no protocol | NA | NA |  | NI |  | PN |  | Some concerns |
| **Li M 2000** | Y | Y |  | NI | no protocol | NA | NA |  | PY |  | NA |  | Some concerns |

**Additional file 4-3 The details of risk of bias assessment-Domain 3. Missing outcome data**

| **Study ID** | **3.1** | **Note for 3.1** | **3.2** | **Note for 3.2** | **3.3** | **3.4** | **Note for 3.3&3.4** | **3.0 Assessor's judgement** |
| --- | --- | --- | --- | --- | --- | --- | --- | --- |
| **Jiang F 2009** | NI |  | PY |  | NA | NA | There may be an unknown missing data, due to the lake of protocol, but given that clinical efficacy is generally better, it is unlikely that the absence of truth values will affect the results | Low |
| **Meng L 2006** | NI |  | PY |  | NA | NA | Same as Jiang F 2009 | Low |
| **Yu F 2013** | PY |  | NA |  | NA | NA | Same as Jiang F 2009 | Low |
| **Duan XW 2011** | PY |  | NA |  | NA | NA | Same as Jiang F 2009 | Low |
| **Zeng L 2005** | PY |  | NA |  | NA | NA | Same as Jiang F 2009 | Low |
| **Liang FL 2012** | NI |  | PY |  | NA | NA | Same as Jiang F 2009 | Low |
| **Wang Q 2014** | NI |  | PY |  | NA | NA | Same as Jiang F 2009 | Low |
| **Shao Z 2011** | PY |  | NA |  | NA | NA | Same as Jiang F 2009 | Low |
| **Pan HR 2010** | NI |  | PY |  | NA | NA | Same as Jiang F 2009 | Low |
| **Wu JL 2011** | PY |  | NA |  | NA | NA | Same as Jiang F 2009 | Low |
| **Liu ZW 2013** | PY |  | NA |  | NA | NA | Same as Jiang F 2009 | Low |
| **Li M 2000** | PY |  | NA |  | NA | NA | Same as Jiang F 2009 | Low |

**Additional file 4-4 The details of risk of bias assessment- Domain 4. Measurement of the outcome**

| **Study ID** | **4.1** | **Note for 4.1** | **4.2** | **Note for 4.2** | **4.3** | **Note for 4.3** | **4.4** | **Note for 4.4&4.5** | **4.5** | **4.0 Assessor's Judgement** |
| --- | --- | --- | --- | --- | --- | --- | --- | --- | --- | --- |
| **Jiang F 2009** | N |  | PN |  | PY | participant-reported outcomes | PY |  | PN | Some concerns |
| **Meng L 2006** | PN |  | PN |  | PY | Same as Jiang F 2009 | PY |  | PN | Some concerns |
| **Yu F 2013** | PN |  | N |  | PY |  | PN |  | NA | Low |
| **Duan XW 2011** | PN |  | PN |  | Y |  | PN |  | NA | Low |
| **Zeng L 2005** | PN |  | PN |  | PY |  | PN |  | NA | Low |
| **Liang FL 2012** | PN |  | PN |  | Y |  | PN |  | NA | Low |
| **Wang Q 2014** | PN |  | PN |  | PY |  | PN |  | NA | Low |
| **Shao Z 2011** | PY |  | PN |  | NA | participant-reported outcomes | NA | no objective outcomes | NA | High |
| **Pan HR 2010** | PN |  | PN |  | PY | participant-reported outcomes | PY | no objective outcomes | PY | High |
| **Wu JL 2011** | PY |  | PN |  | NA | participant-reported outcomes | NA | no objective outcomes | NA | High |
| **Liu ZW 2013** | PY |  | PN |  | NA | participant-reported outcomes | NA | no objective outcomes | NA | High |
| **Li M 2000** | PN |  | PN |  | PY | participant-reported outcomes | PY | no objective outcomes | PY | High |

**Additional file 4-5 The details of risk of bias assessment- Domain 5. Selection of the reported result**

| **Study ID** | **5.1** | **Note for 5.1** | **5.2** | **Note for 5.2** | **5.3** | **Note for 5.3** | **5.0 Assessor's Judgement** |
| --- | --- | --- | --- | --- | --- | --- | --- |
| **Jiang F 2009** | NI | no protocol | NI |  | NI |  | Some concerns |
| **Meng L 2006** | NI | no protocol | NI |  | NI |  | Some concerns |
| **Yu F 2013** | NI | no protocol | NI |  | NI |  | Some concerns |
| **Duan XW 2011** | NI | no protocol | NI |  | NI |  | Some concerns |
| **Zeng L 2005** | NI | no protocol | NI |  | NI |  | Some concerns |
| **Liang FL 2012** | NI | no protocol | NI |  | NI |  | Some concerns |
| **Wang Q 2014** | NI | no protocol | NI |  | NI |  | Some concerns |
| **Shao Z 2011** | NI | no protocol | NI |  | NI |  | Some concerns |
| **Pan HR 2010** | NI | no protocol | NI |  | NI |  | Some concerns |
| **Wu JL 2011** | NI | no protocol | NI |  | NI |  | Some concerns |
| **Liu ZW 2013** | NI | no protocol | NI |  | NI |  | Some concerns |
| **Li M 2000** | NI | no protocol | NI |  | NI |  | Some concerns |
